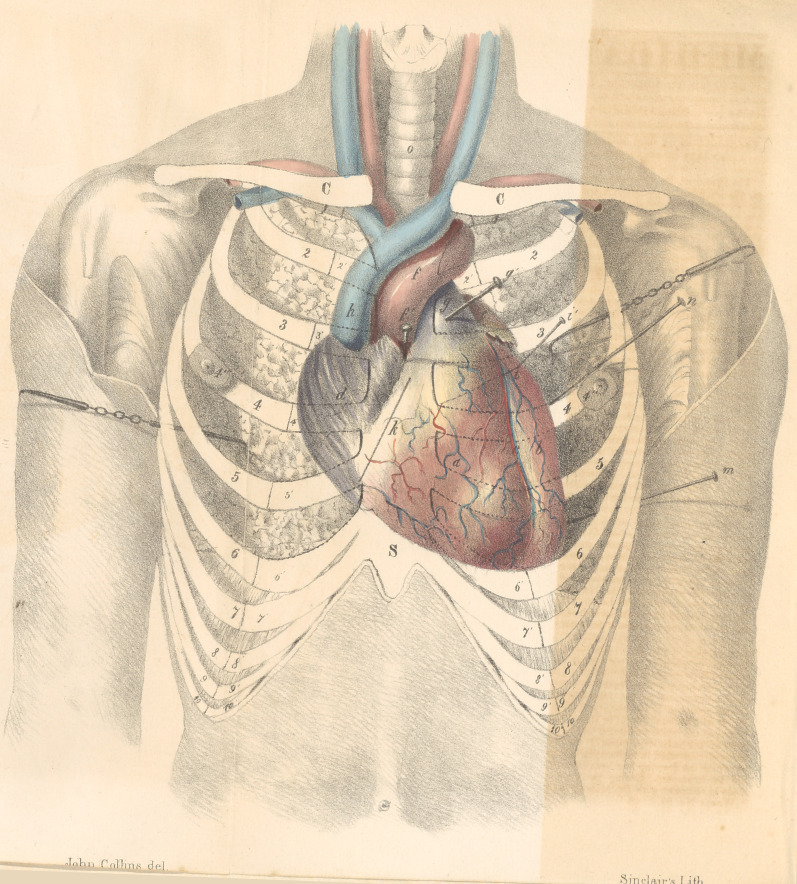# Transactions of the Pathological Society of Philadelphia

**Published:** 1840-04-04

**Authors:** 


// MEDICAL EXAMINER.

DEVOTED TO MEDICINE, SURGERY, AND THE COLLATERAL SCIENCES.

No. 14.] PHILADELPHIA, SATURDAY, APRIL 4, 1840. [Vol. III.

### TRANSACTIONS OF THE PATHOLOGICAL SOCIETY OF PHILADELPHIA.

*March 30th*, 1840.

The President, Dr. Gerhard, in the Chair.

### *On the situation of the Heart--with a plate.*

By C. W. Pennock, M. D.

Although the structure of the heart is as well known as that of any organ of the body, yet its precise situation and its anatomical relations rarely receive from the anatomical student that attention which the importance of the subject demands. Of this fact I am daily more and more convinced. Well instructed physicians, unaccustomed to examine the heart *in situ*, and misled by the terms of "the right and left sides," are apt to imagine and assign situations to the different parts of the central organ of the circulation very different from the reality. One of the most general errors is, that of imagining that the ascending aorta and its semilunar valves are placed to the *left* of the pulmonary artery. The most cursory glance at the heart while in the thorax, would correct this error; it would then be seen that the aorta and its valves lie to the *right* of the pulmonary artery. Another mistake consists in supposing that the anterior surface of the heart on the left of the sternum is formed exclusively by the left cavities of that organ. Inspection of the heart in place, would show that a large proportion of this surface is due to the right ventricle.

The experimental researches of Hope, and others, on the heart's action, having settled many of the formerly mooted points on that subject, the diagnosis of cardiac diseases has been rendered much more certain. But to render these physiological researches available in the diagnosis of disease, it is all important that an exact knowledge should be had of the precise situation of the different valves, and of the several compartments of the heart.

Anxious to aid in the investigation of cardiac diseases, it has occurred to me, that a delineation of the heart in its natural position, would not be unacceptable to the profession. This drawing has been executed with great care and exactness by Mr. John Collins, and is now presented to the society, in the hope that it will be found useful in aiding the recollection of the relative situation of the several parts of the heart with those of the thoracic parietes. A word in explanation of the plan by which the drawing has been effected, may not be improper. During the experiments made in company with Dr. Moore, we observed, that the only fixed point of the heart was at the valves of the aorta, and that those of the pulmonary artery being slightly moveable, revolved partially around them. Repeated observation having proved to me that the aortic valves are pierced, if needles be introduced perpendicular to the plane of the sternum, through the middle of that bone opposite the middle of the cartilages of the third ribs,—and that wires passed perpendicularly to the plane of the thorax, half way between the cartilages of the second and third ribs, half an inch from the left margin of the sternum, pass through the valves of the pulmonary artery, we inserted needles at these points, which served as marks and guides, in the delineation of the heart, when we removed the anterior parietes of the chest. By measuring from these fixed points, we were enabled to mark the relative situation of every part of the heart with rigid accuracy.

Upon reference to the drawing, it will be seen, as previously stated, that the *valves of the aorta* lie beneath the middle of the sternum opposite the lower edge of the cartilages of the third ribs; that the *valves of the pulmonary artery* are more superficial, and are placed to the left and about half an inch above. *The aorta* from its origin, curves upwards towards the right, extending between the cartilages of the second and third ribs slightly beyond the right margin of the sternum; at the lower margin of the second cartilage, the arch of the aorta commences and inclines to the left, crossing the pulmonary artery where it lies beneath the left second rib, and ascending as high as the first rib, turns downwards. The *pulmonary artery*, from its origin in contact with the sternum, commences at its left margin, where it is joined by the cartilage of the third rib, bulges at the interspace between the second and third cartilages close to the sternum, and dips beneath the aorta opposite the junction of the second cartilage and sternum.

The right divisions of the heart being most superficial, form the greater part of the anterior surface; the *right auricle* reaches from the cartilages of the third rib, to that of the sixth, and between the third and fourth, where its breadth is the greatest, it extends laterally near two inches to the right of the sternum. About one-third of the right ventricle lies beneath the sternum, the remaining two-thirds being to the left of that bone; the *septum* between the ventricles coincides with the osseous extremities of the third, fourth and fifth ribs, and on the fourth rib, is midway between the left margin of the sternum and nipple. A

small part, say one-fourth, of the left ventricle presents anteriorly; and when the lungs are separated, a portion of the left auricle is visible between the second and third left ribs, two inches from the left margin of the sternum. With the exception of these portions, the whole of the left ventricle and auricle lie posteriorly to the right ventricle; and the entire left divisions, with the exception of a small portion of the base connected with the pulmonary valves of the aorta, lie on the left of the sternum.

The heart being moveable, the tricuspid and mitral valves necessarily change their relative position with the parietes of the thorax, with every change of posture of the body. When examined in the dead body, the normal situation of these valves is as follows: the *tricuspid valve* extends obliquely downwards from a point in the middle of the sternum, immediately below the the third rib, to the right edge of the sternum, at the lower margin of the cartilage of the fifth rib; the *mitral valve* commences beneath the lower margin of the left third rib, near the junction of its cartilage with its osseous extremity, (two and a half to three inches to the left of the sternum,) and runs slightly downwards, terminating opposite the left margin of the sternum, where it is joined by the cartilage of the fourth rib.*

The apex of the heart, when an individual is standing erect, beats between the fifth and sixth left ribs, about two inches below the nipple, and one inch on its sternal side. But as the heart is attached only at its base by the large blood vessels, "the body of that organ is not fixed in relation to the walls of the chest, but hangs in a certain degree loose," and liable to displacement by change of posture, and by the motions of the chest. Hence, the pulsations of the apex are felt at different points of the chest, and the impulse is affected by the stage of the respiratory act. During full inspiration, the impulse of a healthy heart is scarcely perceptible, but upon expiration, and especially, if, at the same time, the body be bent forward, the cardiac pulsations become very forcible.

I have frequently made observations on the influence of position upon the impulse of the heart. During expiration, when the body is lying on the back, the heart beats at the upper part of the left fifth rib, an inch below the nipple, and one inch to the right of it. Lying on the left side, the impulse is felt at the lower margin of the fifth rib, one inch below the nipple, and an inch and a half to the left of it; if the examination be made while lying on the right side, the impulse is rarely felt. When standing, the body being bent forward, the impulse is felt during expiration one and a half inches below the nipple; bending backwards, impulse observed between the fourth and fifth ribs, half an inch below the nipple, and one inch to the right of it; inclining the body towards the left, throws the apex between the fifth and sixth ribs, two inches below the nipple, and one and a half inches to the left of it; while bending towards the right, causes the impulse to be felt two inches on the sternal side of the nipple.

Effusions or tumours in the abdomen by elevating the diaphragm, displace the heart, and cause the impulse to be felt in various unusual positions; within a month, I have made a post-mortem examination of a patient who died from ascites, in which case, the abdominal fluid pressing upon the diaphragm, had driven the apex *to the third rib, one inch above* the left nipple, at which point the impulse was felt during life.

*Explanation of the Plate.*

The heart is represented with the pericardium removed—the lungs drawn backwards by hooks, leaving its entire anterior surface exposed—the cartilages and ribs in front of it, indicated by dotted lines.

S, Outline of the Sternum.
C, Clavicle.
1, 2, 3, 4, 5, 6, &c., The ribs.
1′, 2′, 3′, 4′, 5′, &c., The cartilages of ribs.
4″, Right and left nipples.
*a*, Right ventricle.
*b*, Left do.
*c*, Septum between the ventricles.
*d*, Right auricle.
*e*, Left auricle.
*f*, The aorta; *f*′, needle introduced through middle of sternum, perpendicular to its plane, opposite cartilages of third rib, passing into the aortic valves.
*g*, The pulmonary artery; *g*′, needle introduced between the second and third cartilage half an inch to the left of the sternum, (perpendicular to the plane of the thorax,) passing into the valves of the pulmonary artery.
*h*, Vena cava descendens.
*i*, Line of direction of the mitral valve; the dotted portion is that part of it posterior to the right ventricle.
*i*′, Needle introduced perpendicular to the plane of the thorax, three inches from the left margin of the sternum, at the lower edge of the third rib, and passing in the mitral valve at its extreme left.
*k*, Line of tricuspid valve.
*m*, *n*, Needles introduced perpendicular to thorax, at points where the dulness of percussion of the heart ceases, and which being projected, pass to the borders of that organ.
*o*, Trachea.

*Note.*—By the expression "perpendicular to the plane of the thorax," used in the preceding

* In ausculting the heart, the points mentioned as indicating the situation of the auriculo-ventricular and semilunar valves, are those to which the stethoscope should be applied, in the investigation of the character of the sounds generated at the respective valves.

explanation, is meant, lines passing at right angles to the tangents of the various curved surfaces existing at different points of the chest.

## Figures and Tables

**Figure f1:**