# Error

**Published:** 1840-04-18

**Authors:** 


Error.—In Dr. Pennock's paper on the situation of the heart, the lateral extent of the right auricle between third and fourth cartilages, should have been stated as being one inch and one third to the right of the sternum, instead of two inches.

